# Determining the Dose of Radiation and Radurisation Effects on the Antioxidant Activity of Fish and the Thermophysical Characteristics of Fish Muscle Tissue

**DOI:** 10.3390/foods8040130

**Published:** 2019-04-18

**Authors:** Roza T. Timakova, Sergey L. Tikhonov, Nataliya V. Tikhonova, Sergey V. Shikhalev

**Affiliations:** Institute of Trade, Food Technology and Service, Ural State Economic University, 8 Marta St/Narodnoy voli, 62/45, Ekaterinburg 620144, Russia; trt64@mail.ru (R.T.T.); tihonov75@bk.ru (S.L.T.); sershih@rambler.ru (S.V.S.)

**Keywords:** fish, EPR spectrum, thermophysical properties

## Abstract

The aim of this article is to develop a method for determining the dose of radiation in the processing of chilled fish and its effect on the antioxidant activity and thermophysical characteristics of muscle tissue. Radiation processing of chilled fish was performed using a linear electron accelerator model LEA-10-10S2. The dose of radiation was determined by the method of electron-paramagnetic resonance. After being treated with ionizing radiation, the cooled fish meets the requirements of the technical regulations of TR TU 021/2011 “On food safety” and TR EAEU 040/2016 “On the safety of fish and fish products”. As a result of our research, a correlation was established between the area of the Electron Paramagnetic Resonance (EPR) signal and the absorbed dose of the radiation dose. We proved a decrease in the antioxidant activity of muscle tissue of fish with an increase in radiation dose. It is established that radiation treatment of chilled fish affects the thermophysical properties of muscle tissue.

## 1. Introduction

Defrosted fish is a perishable food product and its shelf life is determined by the development of microorganisms, the action of tissue enzymes and the resistance of fat to oxidative deterioration. Thus, defrosted fish is characterized by high microbial contamination (up to 10^2^–10^4^ bacteria per 1 cm² of surface) due to the penetration of microorganisms from the gastrointestinal tract into muscle tissue with favorable conditions for the development of microflora, in particular, with a high value of 6.3–6.6 pH and water activity [1]. Fermentation such as, for example, bacterial deaminase leads to the breakdown of fish protein into free amino acids and their further autolysis with the formation of decomposition products, which worsen the organoleptic characteristics of the product. Fish contains easily digestible fats with a high content of polyunsaturated fatty acids, but at the same time, the presence of fatty acids in fish causes the rapid development of oxidative damage.

It should be noted, that thermophysical characteristics play some role for the conservation of freshly caught fish, i.e., the ability to conduct and accumulate heat/cold. The thermophysical properties of fish muscle tissue depend on its chemical composition, muscle tissue density, etc.

Thus, taking into account all the named factors that have a negative effect on fish shelf life, the development of methods for increasing fish shelf life is one of the promising areas of research in storage technology.

A promising physical way to increase the shelf life of defrosted fish is to treat it with ionizing radiation. According to GOST 34154-2017 “Standard guide for irradiation of fish and aquatic invertebrates used as food to control pathogens and spoilage microorganisms”, the use of radiation storage technologies ensures the microbiological safety of fish.

According to the assumptions, the market of irradiated raw food materials and food products may reach $ 4.8 billion by year 2020 and up to $ 10.9 billion by year 2030. At the same time, up to 70% of food irradiation centers are located in the USA and China [2].

The use of ionizing radiation contributes to the destruction of parasitic protozoa and worms that are dangerous to human health, and of microorganisms that cause food spoilage [3].

It was proved that the treatment of fish fillets with doses of gamma-radiation from 1 kGy to 4 kGy is more effective compared to the traditional methods of preservation due to the destruction of pathogenic and conditionally pathogenic microflora. It was established that radiation dose of 3 kGy is sufficient to destroy *S. aureus* and *E. coli*; at radiation dose of 4 kGy *Listeria* monocytogenes is destroyed without changing the physicochemical properties of the fish [4]. The use of gamma radiation and X-rays in order to destroy pathogenic strains of bacteria, such as vibriosin particular, in raw food fish materials is an alternative to heat treatment [5].

However, radiation treatment to high doses (over 4.5 kGy) can lead to the development of oxidative damage of fish; to the reduction of concentration of fatty acids; and to the increased synthesis of thiobarbituricacid and, as a result, it can lead to the formation of radiolysis products and the destruction of thiamine (B1) while maintaining riboflavin (B2) [6,7,8].

Codex Alimentarius has established a maximum dose of absorbed radiation of up to 10 kGy. At the same time, in the countries of America (USA, Canada, Brazil), the maximum permissible dose for processing fish products is 2.2 kGy, in the EU countries (England, France, The Netherlands)—3.0 kGy [9].

At the same time, there is a number of unresolved issues that remain in the technology of processing and defrosted fish with ionizing radiation: technological parameters of processing ionizing radiation of fish are not established to ensure high quality characteristics during storage, including preserving its antioxidant activity and preventing the development of enzymatic and oxidative damage; the effect of ionizing radiation on the thermal conductivity of irradiated defrosted fish has not been studied, there are no regulations that can be used to identify irradiated fish products.

In this regard, the aim of the present work is to develop a method for determining the dose of ionizing radiation during the processing of defrosted fish and its effect on the antioxidant activity and the thermophysical characteristics of muscle tissue.

## 2. Materials and Methods

For experimental studies, rainbow trout (*Salmo irideus*) cooled and gutted with a head weighing between 700 g and 1000 g, up to 25 cm long, and up to 2 cm thick, taken in the third quarter was used. Studies were performed after two days of storing fish in the refrigerator after catching at a temperature from 0 to 2.

Samples of the first group trout (control) were not treated with ionizing radiation; the samples of the second experimental group were treated with an ionizing radiation dose of 1 kGy, the third—2 kGy, the fourth—3 kGy, the fifth—4 kGy. There were five fish samples in each group (*n* = 5). The choice of radiation doses was performed based on the analysis of scientific and technical information, in which it is recommended to irradiate fish with doses from 1 to 2.5 kGy [10,11].

The sampling of cooled fish was carried out in accordance with the requirements of GOST 7631-2008 “Fish, non-fish objects and products made of it. Methods for the determination of organoleptic and physico-chemical parameters”. The ionizing radiation of the studied samples of chilled trout gutted with the head was performed at the Radiation Sterilization Center (RSC) of the Ural Federal University named after the first President of Russia B.N. Yeltsin. A linear electron accelerator model UELR-10-10C2 with energy up to 10 MeV was used for irradiation. The study of irradiated cooled fish was carried out on a portable automated Electron Paramagnetic Resonance (EPR) spectrometer of the X range of the Labrador Expert brand at a microwave frequency of 9200 MHz in the magnetic field range from 3.000 to 3.500 Gs. The main characteristics of the electron beam are indicated in Table 1. To control the dose after irradiation, the method of photo-spectroscopy was used by means of measuring optical density of the irradiated polymer film on the spectrophotometer at a wavelength of 512 nm.

The choice of the dosimetry system (equipment) for determination of the absorbed dose is associated with the fact that one of the most effective and practicable methods of investigation and identification of radicals, induced by radiation, is electron paramagnetic resonance spectroscopy. Bone tissue density, structure and composition are heterogeneous, the variations of the absorbed dose differ on the surface, inside and in different parts of the sample. Therefore, the use of a precise EPR method of the average sample allows to obtain reliable and repeatable results. EPR spectra were measured and processed using a specialized computer program for an EPR spectrometer. The main parameters of the radiation signal were determined: g-factor, amplitude, width and area of the EPR spectrum.

The dose of ionizing radiation of radiation-treated fish was determined on bone tissue samples (BTS). For this purpose, a special sample preparation was carried out. The basic principles of sample preparation are based on the main provisions of GOST R 52529-2006 Meat and Meat Products. Electron Paramagnetic Resonance Method for Detecting Radiation-Treated Meat and Meat Products Containing Bone Tissue.

The technique of preparing bone tissue samples (BTS) of fish differs from the technique of GOST R 52529-2006, increasing the duration of bone tissue drying up to 30 h, which allows obtaining stable EPR spectra and, accordingly, reliable results.

Fish bones are cleaned from muscle tissue Then it is dried in a dryer at a temperature of 39–40 °C during 24–30 h until the residual moisture of 3–4%, cooled, incubated at a room temperature during 30–40 min and crushed to the size of separate fragments up to 0.5 × 0.5 × 0.5 mm with a total mass of at least 0.05 g.

The tested BTS are weighed with the accuracy of the third decimal place, put in labeled quartz ampoules with a height of 10.0 ± 0.5 mm, and placed in the working area of the resonator at a fixed depth.

The authors of the sources [12] state that the establishment of the fact of irradiation with EPR-method is possible due to the presence of long-lasting free radicals, especially of the radical anions CO_2_^−^, CO_3_^3−^, SO_2_^−^ and SO_3_^−^. The authors of the source [13] used EPR for detection of irradiation in the bones of agricultural animals and poultry, fish and mollusc shell, and found that the EPR signals are stable in mammal bones and mollusc shells.

In the experiment, the value used to estimate the degree of ionizing radiation impact on the sample was taken as the radiation dose; as the absorbed dose (the amount of ionizing radiation energy absorbed by the samples).

Antioxidant activity (AOA) was determined by a potentiometric method using an MPA-1 analyzer. In order to determine the thermophysical characteristics of the trout, the test samples were ground before and after the treatment with ionizing radiation to a stuffed state (a mince state) in accordance with the requirements of GOST R 55505-2013 Frozen Minced Fish Food. Technical Specifications.

**Figure 1:**
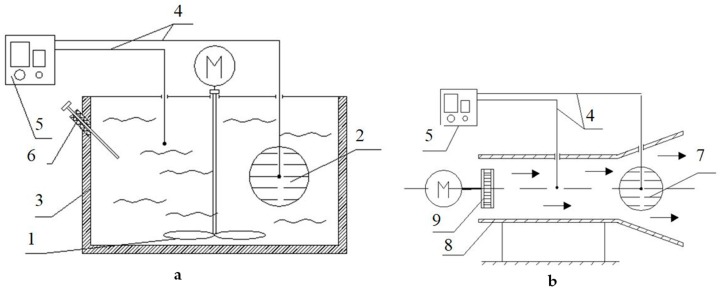
Setup for determining the thermal diffusivity and thermal conductivity coefficient of minced fish; (**a**)—water thermostat and (**b**)—air thermostat. 1—stirrer; 2—α-calorimeter; 3—liquid thermostat; 4—chromel-copel thermocouples; 5—microvoltmeter; 6—checking thermocouple; 7—λ-calorimeter; 8—airswitch; 9—fan.

The thermal diffusivity and thermal conductivity coefficient of minced fish was determined using the first regular mode method proposed by G.М. Kondratyev [14]. For this purpose, a test setup shown in Figure 1 was developed.

In order to find the thermal diffusivity, the test-bed consisted of a liquid thermostat 3 (Figure 1a), which allows to maintain a constant temperature and ensure that the condition α tends to infinity, α-calorimeter 2 in the form of a spherical copper shell of 60 mm in diameter with the substance under the test (minced fish), and mixing device 1.

The thermal conductivity coefficient was found using a λ-calorimeter 7 (Figure 1b) in the form of a spherical copper shell with the test substance and using an airswitch (8) with a fan (9).

In the process of cooling, the temperature difference between the product in the calorimeter and the cooling medium was recorded with a differential chromel-copel thermocouple 4 and a noise-proof PMPT-1 DC microvoltmeter (5), whose readings were recorded with a KSP-4 potentiometer (not shown on the test-bed). The temperature measurement error did not exceed 0.01 °C.

The thermal diffusivity and thermal conductivity coefficient were found by experimentally determining the rate of cooling of fish mince samples on α-calorimeter and λ-calorimeter. The specific heat capacity of the trout minced fish was determined by comparative cooling of the sample and a geometrically similar reference (fish meal) by removing the process cooling curves.

All tests were performed in fivefold replication. The results were processed by the method of variation statistics using the Microsoft Excel computer program with Student’s t-test. Confidence level was 0.99 and 0.999. The research results are analyzed with the method of analysis of variance with the use of Student coefficient.

## 3. Results

**Figure 2:**
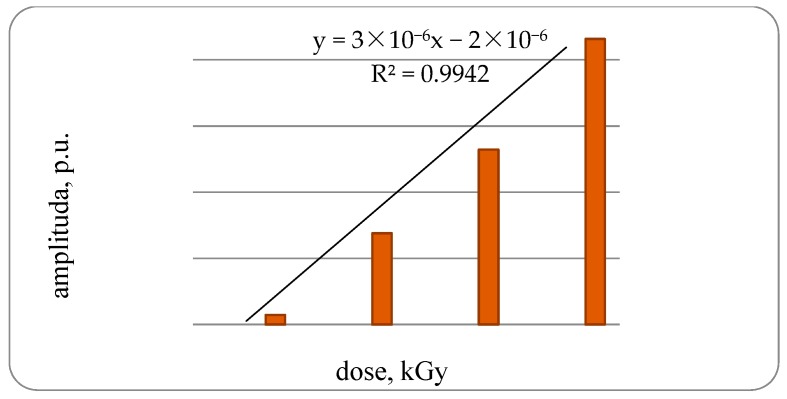
Amplitude of Electron Paramagnetic Resonance (EPR) signals samples of bone tissue of rainbow-cooled trout irradiated with different doses.

It was established that in the control samples of fish the EPR spectrum is not pronounced. The amplitude of the EPR signal samples of bone tissue of a trout under irradiation with a dose of 1 kGy is 1.29 × 10^−6^ rel. units, increasing the dose to 2 kGy amplitude increases to 3.76 × 10^−6^ rel. units or 2.9-times the samples of bone tissue of trout irradiated with doses of 1 kGy, with an increase in the dose up to 3 kGy, respectively, to 6.29 × 10^−6^ rel. units or 1.7-times, compared with the third group. With an increase in the radiation dose to 4 kGy, an amplitude increase of 7.5-times was found compared with bone samples irradiated with a dose of 1 kGy, with a high degree of correlation dependence—0.9942 (Figure 2).

**Figure 3:**
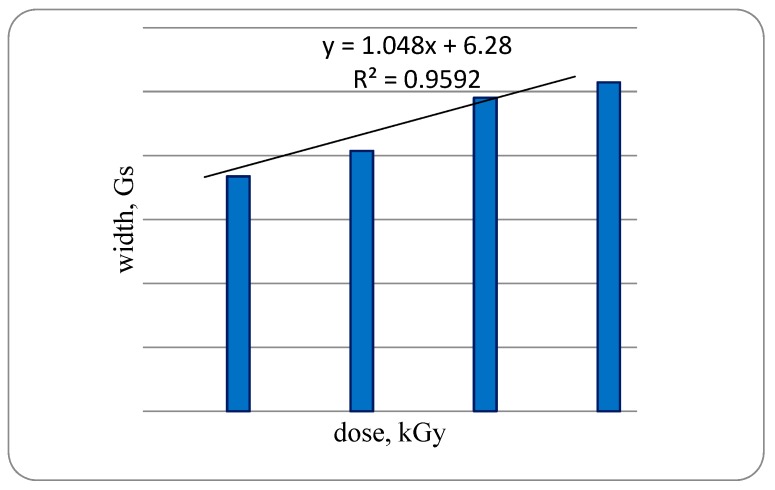
Width of EPR signals samples of bone tissue of rainbow-cooled trout irradiated with different doses.

The width of the samples of bone tissue signal of a trout irradiated with a dose of 1 kGy is 7.5 G, while increasing the dose to 2 kGy increases to 8.15 G or 10.9% compared with OCT of a trout irradiated with 1 kGy when increasing the radiation dose up to 3 kGy, the signal width is increased by 20.4% compared with trout specimens irradiated with a dose of 2 kGy. With an increase in radiation dose of up to 4 kGy, an increase in the signal width by 40.0% was established as compared with bone samples irradiated with a dose of 1 kGy, with a high degree of dependence—0.9592 (Figure 3).

**Figure 4:**
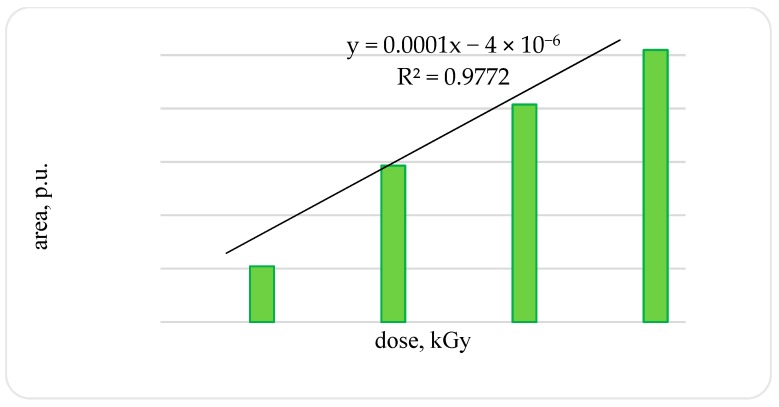
Area of EPR signals samples of bone tissue of rainbow-cooled trout irradiated with different doses.

An increase in the amplitude and width of the signal leads to an increase in the signal area: in the OCT of a trout irradiated with a dose of 2 kGy compared to the OCT of a trout irradiated with a dose of 1 kGy, by 2.8-times; in OCT of a trout irradiated with a dose of 3 kGy compared with OCT of a trout irradiated with a dose of 2 kGy, by 1.4-times. After OCT irradiation of a trout with a dose of 4 kGy compared with samples irradiated with a dose of 1 kGy, an increase in the signal area by 4.9-times was established (confidence level 0.99) (Figure 4).

**Figure 5:**
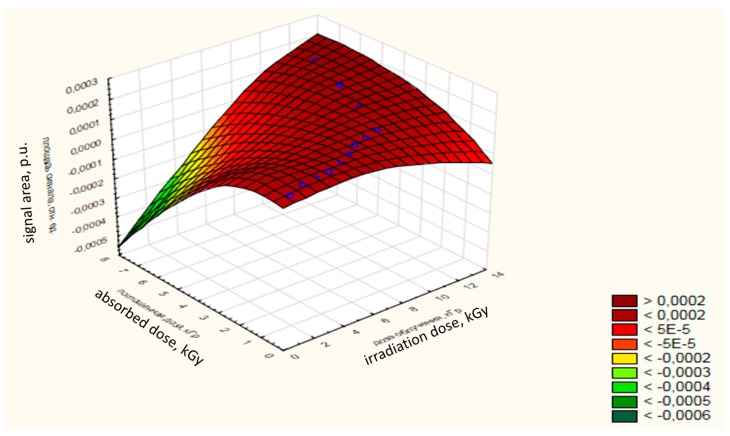
Dependence of change in the absorbed dose (Y) and irradiation dose (X_1)_, as well as signal area (X_2_) for OCT.

The ratio of the absorbed dose to the dose of radiation and the area of the EPR signal of samples of chilled trout is 0.95. The dependence of the absorbed dose-change on the radiation dose and on the area of the signal for OCT of trout is shown in Figure 5. The absorbed dose with an increase in the dose of radiation tends to increase, as evidenced by the area under the signal of the EPR spectrum.

According to [15,16], the formation of free radicals under the action of ionizing radiation on food products is noted.

Since the test object by EPR spectroscopy is the steady-state concentration of free radicals [17], therefore, our data on changes in the structure of the EPR spectra (width, height, and peak area) with increasing radiation dose are consistent with the regularities of EPR signal generation described in [17], in which the author claims that one of the most informative characteristics of the EPR spectra is the hyperfine structure of the spectrum (HFS), which is determined by the interaction of the unpaired electron with the magnetic nuclei that make up the radical, and the HFS analysis gives information about the distribution unpaired electron in a molecule (spin density) [17].

**Figure 6:**
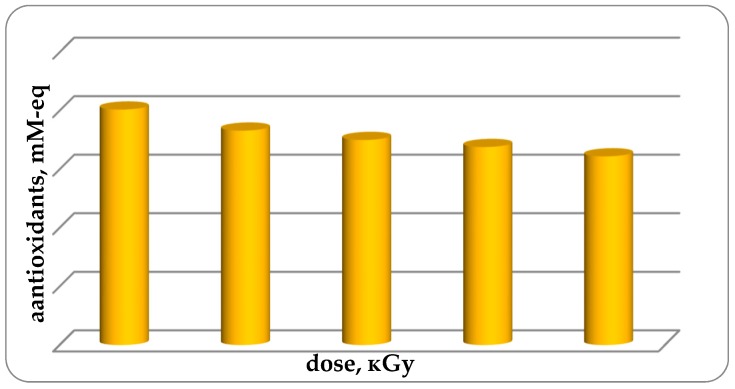
AOA fish samples (*p* ≤ 0.01).

Studies of antioxidant activity (AOA) of fish muscle tissue have been conducted. It was established that the AOA of non-irradiated trout samples (group 1 control) is 0.201 ± 0.085 mM-eq. With an increase in the radiation dose from 1 kGy to 4 kGy, the AOA in samples of chilled trout muscle tissue decreases from 0.201 ± 0.022 for unirradiated samples to 0.161 ± 0.011 mM-eq for samples. irradiated with a dose of 4 kGy, or 19.9% with a high degree of confidence (up to 0.97) (Figure 6). Possible reduction of AOA is due to the fact that antioxidants interact with free active radicals and form low-active radicals. The data obtained are consistent and compared with the test results [18], measuring the EPR spectra obtained on a JES Fa 300 spectrometer (JEOL, Tokyo, Japan) in the X-band of lyophilized organs of laboratory animals subjected to electromagnetic field treatment (EMF) in the range of 3–16 Hz, 30 µT magnetic induction for 1 h. It is known that the effect of EMF, as well as ionizing radiation, on biological objects can promote the formation of free radicals and therefore reduces the antioxidant properties of the test object [19]. According to the data obtained by EPR spectroscopy in rats exposed to EMF, the formation of free radicals was observed—an increase in the number of paramagnetic centers by 16–19%. In addition, the treatment of rats with a low-frequency electromagnetic field had a negative effect on the prooxidant-antioxidant system of the body by increasing the intensity of free-radical oxidation processes [18] that is consistent with a decrease in the antioxidant activity (AOA) of cooled fish samples treated with ionizing radiation (Figure 6).

**Figure 7:**
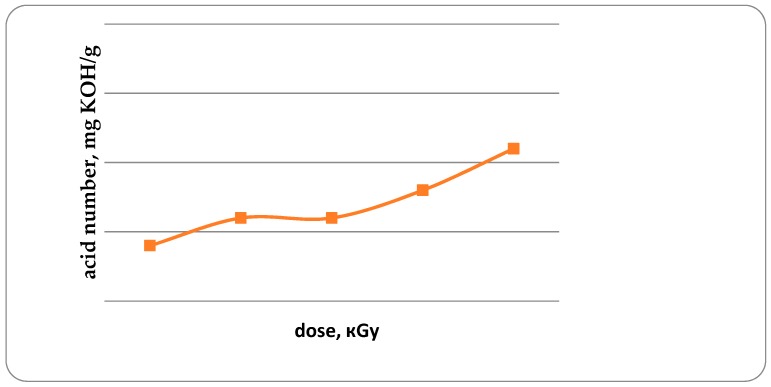
Acid number in trout samples, mg KOH/g (*p* ≤ 0.01).

**Figure 8:**
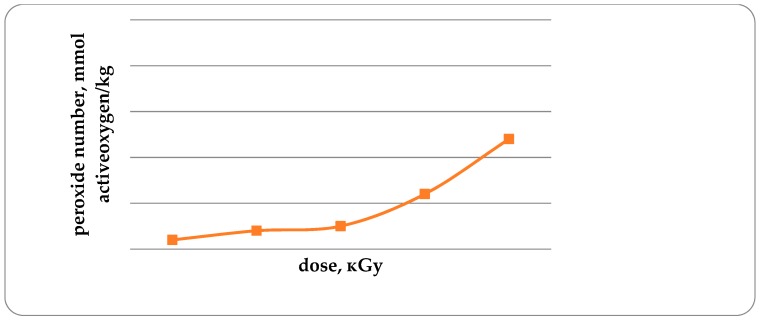
Peroxide number in trout samples, mmol of active oxygen/kg (*p* ≤ 0.01).

The acid and peroxide numbers of fat are the important indicators characterizing the development of oxidative damage to fish [20]. Acid number (AN) in unirradiated trout samples, and in irradiated trout samples with doses from 1 kGy to 4 kGy is 0.14; 0.16; 0.16; 0.18 and 0.21 mg KOH/g, respectively (Figure 7). It has been established that with an increase in the radiation dose from 1 to 4 kGy, the AN increases by 50%. Similar data were obtained for the study of peroxide values (Figure 8).

An increase in the acid and peroxide numbers of trout lipids with an increase in the radiation dose is a confirmation of a decrease in the concentration of antioxidants (Figure 6). The peroxide number in unirradiated and irradiated trout samples with doses from 1 kGy to 4 kGy is 0.32; 0.34; 0.35; 0.42 and 0.54, mmol of active oxygen/kg. Increasing the radiation dose from 1 to 4 kGy leads to an increase in fish lipid peroxide by 68.8%. It should be noted that the acid number and peroxide number of lipids of non-irradiated fish are lower by 14.3 and 6.3%, respectively, in comparison with the irradiated fish dose of 1 kGy.

**Figure 9:**
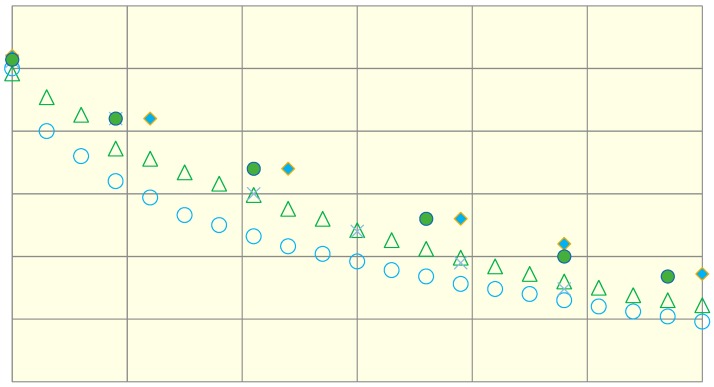
The cooling rate of the samples of minced fish in air thermostat: **⚪**—1 (dose 0 kGy); ♦—2 (dose 1 kGy); ●—3 (dose 2 kGy); **∆**—4 (dose 3 kGy); ×—5 (dose 4 kGy).

The results of studies of thermophysical properties are presented in Table 1 and in Figure 9.

It was established that when cooling minced trout meat in an air thermostat, the cooling rate of samples of the first control group was lower than in the experimental groups of samples irradiated with different doses, that is consistent with the results obtained [21]. It was found that the rate of cooling of control samples and prototypes of the fourth group irradiated with a dose of 3 kGy is comparable. The results indicate that the treatment of minced fish with ionizing radiation at doses of 1, 2, 3 and 4 kGy increases the thermal diffusivity factor of minced fish by 3.8%, 6.4%, 12.8% and 25.6%, respectively.

An analysis of the experimental values of the thermal conductivity coefficient showed that as a result of the processing of fish samples with ionizing radiation, the value of the thermal conductivity coefficient increases. So, in samples of the 5 experimental group it increased by 20.0% compared with untreated samples. The coefficients of the specific heat capacity of muscle samples of radiation-treated trout cooled with increasing radiation dose are comparable to samples of the first control group. The coefficient of the specific heat capacity of fish samples of 2, 3, 4 and 5 groups is lower by 1.8%, 0.9%, 0.7% and 0.6% in comparison with control samples, respectively.

## 4. Discussion

Thus, as a result of this research, it was established that with increasing meat radiation dose from 1 kGy to 4 kGy, the EPR signal parameters significantly increased (signal amplitude by 7.5 times, signal width by 40.0%, signal area by 4.9-times) and absorbed dose by 25.6% (*p* ≤ 0.05). The obtained experimental parameters of the EPR spectrum of irradiated trout allow us to recommend the EPR method for determining the radiation dose and, in general, for the identification of radiation-treated fish. It has been established that the radiation of fish leads to a significant decrease in AOA, which is consistent with the activation of fish lipid peroxidation. The treatment with ionizing radiation of fish leads to changes in its chemical composition and the formation of hydroxyl radicals, hydrated electrons, and hydrogen atoms [22,23], which is consistent with the results of research [24], which state that the presence of free organic radicals in the irradiated food product can cause various reactions. They can reduce minor components, separate hydrogen atoms from thiols and alcohols; interact with oxygen to form peroxide radicals; decay with the formation of new radicals, causing a chain reaction; react with each other through dimerization or disproportionation, forming new products. The primary ionization processes occurring in objects that contain a significant amount of moisture are completed in about 10–100 s, with the formation of molecules of new organic compounds and free organic radicals. These radicals are formed both indirectly, under the influence of hydroxyl radicals, hydrated electrons, and hydrogen atoms, and as a result of the direct action of irradiation.

According to [25], the activity of radicals in irradiated food products depends on the moisture content, temperature, and the presence of oxygen.

The formation of free radicals in the muscular tissue of fish, and, accordingly, an increase in lipid peroxidation products (acid and peroxide number) with an increase in radiation dose is associated with a high content of chemically and mechanically associated water (55–85%) in fish, which is consistent with research [23,24,25,26], claiming that water molecules are excited and ionized to a much greater degree than other components of the food product and form the products of radiolysis of water. Hydroxyl radicals can select hydrogen atoms from carbon–hydrogen bonds in aliphatic compounds, for example, in amino acids, etc., to add hydrogen atoms at the breaking of a hydrogen sulfide bond. Consequently, if there are both aromatic and aliphatic components in the system (for example, in proteins or nucleic acids in fish), some hydroxyl radicals react by the type of attachment, and some by the type of detachment. In any case, the reaction product is an organic free radical.

The other main reactive elements in the irradiated systems containing water, in particular, in our experiment in the irradiated meat, are hydrated electrons. According to [26], they are more selective than hydroxyl radicals; therefore, they do not always interact with the main components of the system but can react with vitamins, pigments, etc. As is well known, fish contains vitamins of groups B, D, PP and pigments. Hydrated electrons in proteins combine with histidine, cysteine, cystine residues and with some other amino acids, but they do not give significant reactions.

Reactions of hydrogen atoms have an intermediary position between the reactions of hydroxyl radicals and hydrated electrons. Like hydroxyl radicals, they can combine with various chemical compounds, although the rate of these reactions is several times lower than hydroxyl radicals. In fish muscle proteins, their main reactions are directed to sulfur-containing and aromatic amino acids.

According to [27], the effect of ionizing radiation on proteins leads to the breaking of hydrogen bonds of the protein molecule with its subsequent expansion, aggregation, or dissociation into smaller elements, or fragmentation. There is an opportunity for reactions of protein radiolysis products with other substances entering the protein environment, with the formation of new compounds not characteristic of specific products. All this leads to the disappearance of the usual properties of the protein: enzymes can lose their activity; pigments change their color, etc. [27], which is consistent with the results of our previous studies 1.

Other products of the radiolysis of amino acids are ammonia, carboxylic acids, amines, and carbon dioxide, resulting from their reductive deamination and decarboxylation. The decay of amino acids, including essential ones, reduces the nutrition value of the irradiated products [28]. In the free state, sulfur-containing amino acids with thiol or disulfide groups are particularly sensitive to ionizing radiation. By the sensitivity to ionizing radiation, aromatic and heterocyclic amino acids can be put in the following line: histidine > phenylalanine > tyrosine > tryptophan, and proline and oxyproline are the most stable with respect to irradiation [27].

As is known, fish contains up to 30% fat in its chemical composition and it is characterized by the presence of important polyunsaturated fatty acids Omega-3 and Omega-6. According to [24], the treatment with ionizing radiation of high-fat foods leads to autoxidative changes leading to the formation of typical oxidation products and non-oxidative radiological changes, resulting in the formation of specific radiolytic compounds. Irradiation accelerates the process of auto-oxidation, especially in the presence of oxygen during and after irradiation. Products found in irradiated fats are identical to those normally found in non-irradiated but oxidized fats. The degree of oxidative changes during irradiation depends on factors that (except for the irradiation dose) are also typical for oxidation without irradiation: temperature, the presence or absence of oxygen, catalysts, antioxidants, fat composition, etc., which is consistent with the results of the peroxide and acid fish fat numbers. Moreover, irradiation leads to a decrease or complete disappearance of polyunsaturated acids, which largely determine the nutrition value of fish fats. According to [29], the consumption of products containing peroxides contributes to the accumulation of toxic substances resulting from the peroxidation of lipids in cells, which leads to a change in the structural and functional properties of the membranes, up to the degradation of their structures, and, as a result, sharp disruption of membrane permeability. The resulting toxic radicals have a damaging effect not only on lipids but also on the proteins of cell membranes; in addition, their toxic effect on the genetic apparatus of the cell has been established.

We also cannot ignore the results of studies [24,25], which state that, under the influence of ionizing radiation, minor amounts of toxic compounds are formed in food products (acetaldehyde, acetone, formaldehyde, formic acid, benzene, toluene, hydrogen sulfide, dimethyl disulfide, etc.), which cannot cause direct poisoning of the human body. However, it must be borne in mind that various radiolysis products are able to interact with each other, with the possible emergence of toxic substances. Moreover, these compounds can accumulate and manifest as chronic poisoning, as well as mutagenic, carcinogenic, or other adverse effects.

Changes in the structure of the compounds that make up the product affect the sensitivity of the enzymes of the digestive tract, reduces its ability to digest and assimilate food. At the same time, from the point of view of the modern theory of metabolic processes, the chemical structure of food components should correspond to the enzyme systems of the body. Its qualitative and quantitative violations are the cause of intolerance and incompatibility of food products, which can manifest themselves in various forms of allergic reactions of the body [28].

The data on food irradiation suggest that our studies of irradiated fish are consistent with the results of [22,23,24,25,26,27,28,29,30], and it can also be assumed that products exposed to ionizing radiation carry a potential hazard to human health.

Irradiation of fish leads to a change in its thermophysical properties—the rate of fish cooling increases, thermal diffusivity and thermal conductivity, and the specific heat capacity irradiated with increasing radiation dose tends to decrease. The results of studies of the thermophysical characteristics of irradiated fish can be taken into account during its heat treatment.

## Figures and Tables

**Table 1:** Parameters of the beam of accelerated electrons of UELR-10-10C2 linear electron accelerator.

Parameter	Characteristic
Maximum energy of accelerated electrons, MeV	10
Maximum average current of dumped electron beam, mA	1
Adjustment range of electron energy, MeV	8–10
Frequency of electronic current impulses sequence, 1/s	50–240
Maximum size of irradiation field at a distance of 100 mm from exhaust foil, mm	600 × 20
Uniformity of irradiation field along the reamer length on the surface of the irradiated cases, %	±5
Frequency of electron beam scanning, Hz	1–3
